# Elevated Serum Lysophosphatidylcholine in Patients with Systemic Lupus Erythematosus Impairs Phagocytosis of Necrotic Cells *In Vitro*

**DOI:** 10.3389/fimmu.2017.01876

**Published:** 2018-01-17

**Authors:** Gerhard E. Grossmayer, Hildegard Keppeler, Sebastian Boeltz, Christina Janko, Jürgen Rech, Martin Herrmann, Kirsten Lauber, Luis E. Muñoz

**Affiliations:** ^1^Department of Internal Medicine 3 – Rheumatology and Immunology, Universitätsklinikum Erlangen, Friedrich-Alexander-Universität Erlangen-Nürnberg, Erlangen, Germany; ^2^Department of Internal Medicine II, University of Tübingen, Tübingen, Germany; ^3^Department of Otorhinolaryngology, Head and Neck Surgery, Universitätsklinikum Erlangen, Else Kröner-Fresenius-Stiftung Professorship, Erlangen, Germany; ^4^Department of Radiation Oncology and Radiotherapy, University Hospital, Ludwig-Maximilians-University, Munich, Germany

**Keywords:** systemic lupus erythematosus, rheumatoid arthritis, efferocytosis, lysophosphatidylcholine, phagocytosis, necrotic cells, serum, clearance deficiency

## Abstract

**Objectives:**

Impaired clearance of dying and dead cells by professional and amateur phagocytes plays a crucial role in the etiology of systemic lupus erythematosus (SLE). While dying, cells expose and release a plethora of eat-me and find-me signals to ensure their timely removal before entering the dangerous stage of secondary necrosis. A well-described chemoattractant for macrophages is dying cell-derived lysophosphatidylcholine (LPC). However, its implications for and/or its association with SLE disease, so far, have not been examined. In the present study, we analyzed the LPC serum concentrations of patients with SLE and rheumatoid arthritis (RA). Subsequently, we examined if and to which extent the measured serum concentrations of LPC and an LPC-rich environment can impact the phagocytosis of necrotic cells.

**Methods:**

Sera from patients with SLE, RA, and normal healthy donors (NHD) were characterized for several parameters, including LPC concentrations. Phagocytosis of dead cells by human macrophages in the presence of SLE and NHD sera was quantified. Additionally, the impact of exogenously added, purified LPC on phagocytosis was analyzed.

**Results:**

Patients with SLE had significantly increased LPC serum levels, and high serum LPC of SLE patients correlated significantly with impaired phagocytosis of dead cells in the presence of heat-inactivated serum. Phagocytosis in the presence of sera from NHD showed no correlation to LPC levels, but exogenous addition of purified LPC in the range as measured in SLE patients’ sera led to a concentration-dependent decrease.

**Conclusion:**

Our data show that high levels of LPC as observed in the sera of SLE patients have a negative impact on the clearance of dead cells by macrophages. Chemoattraction requires a concentration gradient. The higher the LPC concentration surrounding a dying or dead cell, the smaller the achievable gradient upon LPC release will be. Thus, it is feasible to assume that elevated LPC levels can interfere with the build-up of a local LPC gradient during cell death, and hence might play a role in the establishment and/or perpetuation of SLE disease.

## Introduction

The process of apoptotic cell clearance—commonly referred to as efferocytosis—works with an impressively high degree of effectiveness and eliminates billions of dying cells in the context of tissue homeostasis and cell turnover every day (1). It is so efficient that, under physiological conditions, apoptotic corpses are rarely to be found. Defects and perturbations in this highly coordinate process result in the persistence of cellular debris and have been linked to the etiology and pathogenesis of systemic autoimmune diseases such as systemic lupus erythematosus (SLE) (2–5). Chronic, generalized inflammation, and multi-organ damage are the most typical manifestations of SLE (6).

It is widely accepted that uncleared secondary NEcrotic cell-derived material (SNEC) in germinal centers of secondary lymphatic organs serves as survival and proliferation signal for autoreactive B cells, which will then produce antinuclear antibodies (ANAs) in the periphery (7, 8). The co-existence of ANAs and accumulated SNEC leads to the formation of immune complexes in several tissues (9). This favors a pathological, proinflammatory mode of elimination leading to increased organ damage and accumulation of more dying cell debris, thus fueling a self-amplifying vicious circle (10). The result is a systemic type I interferon signature in response to the ANA–SNEC complexes analogous to a viral infection (3, 11).

Clinically, SLE presents as a very heterogeneous autoimmune disease with a broad range of manifestations. The prevalence of 20–150 cases per 100,000 has remained constant in the US in the last 20 years (12–14). Adult women are affected seven times more often than men and their life expectancy is considerable lower than that of the healthy population (15). Typically, the course of disease is characterized by remissions and relapses ranging from mild to severe with the latter ones also being known as flares (16). The classification as SLE of a patient with autoimmune manifestations is usually done by rheumatologists after meeting at least 4 out of 13 clinical and serological criteria of the American College of Rheumatology (17). Although efferocytosis is closely related with the etiopathogenesis of SLE, none of these criteria so far are associated with the clearance of dead and dying cells.

In order to attract phagocytes for their timely removal, dying cells release soluble find-me signals. Apart from nucleotides, the chemokine CX_3_CL1, sphingosine-1-phosphate, and others, lysophosphatidylcholine (LPC) was one of the first dying cell-derived find-me signals that has been identified (3, 18, 19). We and others have shown that the release of LPC during apoptosis is orchestrated by caspase-3-mediated cleavage and activation of calcium-independent phospholipase A_2_ (iPLA_2_), and subsequent involvement of the ATP-binding-cassette transporter ABCA1 (18, 20). Once released, LPC stimulates chemotaxis of monocytes and macrophages with involvement of the G protein-coupled receptor G2A (21–24). Intriguingly, mice with genetic deletion of G2A develop a late-onset multi-organ autoimmune condition, which closely resembles human SLE (25), suggesting that interference with the LPC-G2A axis—presumably *via* disturbed phagocyte recruitment during efferocytosis—favors the development of autoimmunity. Therefore, the question arises if patients with autoimmune diseases display alterations in the LPC–G2A axis. To date, this has not been studied systematically. Previously, we performed transcriptomic profiling analyses in peripheral blood monocytes of SLE patients. G2A was among the examined candidate genes, but we did not detect any significant alterations in the SLE group in comparison to normal healthy donors (NHD) (26).

In the present study, we focused on LPC and analyzed the corresponding serum levels in SLE patients, patients with rheumatoid arthritis (RA), and NHD. We observed significantly increased LPC serum levels in the SLE group and particularly high levels in patients with involvement of vessels or kidneys, respectively. Since this can be a cause as well as a consequence of impaired efferocytosis, we next examined dead cell phagocytosis in the presence of SLE sera and in the presence of NHD sera supplemented with LPC concentrations that have been observed in the SLE group. A significant correlation of elevated LPC levels with impaired phagocytosis of dead cells in the presence of heat-inactivated SLE sera was detected and further confirmed by exogenous addition of purified LPC to NHD sera. Accordingly, we conclude that increased LPC levels can interfere with local dead cell-derived LPC, which functions as a phagocyte attraction signal. This might contribute to the impairment of clearance and consequently to the establishment and/or maintenance of SLE disease.

## Patients and Methods

### Patients

We obtained sera from patients with SLE visiting our inpatient and outpatient departments. Demographic and clinical data available in 50 patients with SLE were summarized together with routine serology in Tables 1 and 2. Serologic parameters were measured in our routine laboratory immediately after blood drawing. Disease activity was recorded according to the SLE Disease Activity Index 2000 (SLEDAI-2K) or to the European Consensus Lupus Activity Measurement (ECLAM). All patients showing disease activity defined as a SLEDAI score of at least 5 or as an ECLAM score of at least 4 were considered as in flare. Organ involvement was determined by the affection of a specific system due to lupus pathology as ascertained by the treating physicians. Sera from further 41 patients with SLE, from 20 patients with RA, and from 25 NHD were used for the measurement of LPC levels (no clinical data available). All patients fulfilled the American College of Rheumatology criteria for the classification of SLE and RA, respectively (17, 27). A written informed consent was obtained from all blood donors and the study received the final approval from the ethics committee of the Friedrich-Alexander University Erlangen (Permit number 52_14 B).

**Table 1:** Demographics of the cohorts studied.

Demographics	Normal healthy donors	Rheumatoid arthritis	Systemic lupus erythematosus
*n*	25	20	50
Sex M/F	13/12	13/7	5/45
Mean age (years)	31.5 ± 7.3	49.8 ± 17.8	43.2 ± 14.0

**Table 2:** Serological and clinical characteristics of the systemic lupus erythematosus cohort.

	In flare	No flare	*p*
**Serology**			
*n*	12	33	
CRP, median in mg/dl (IQR)	1.2 (1.7)	0.4 (1.5)	0.189^a^
C3, median in mg/dl (IQR)	72.5 (37.5)	93.0 (63.0)	0.098^a^
C4, median in mg/dl (IQR)	10.7 (10.2)	14.2 (10.2)	**0.038^a^**
Anti-dsDNA, median in U/ml (IQR)	33.8 (140.2)	11.8 (44.5)	0.097^a^
Antinuclear antibody titer, median^−1^ (IQR)	210.0 (280.0)	320.0 (980.0)	0.518^a^
APS, *n* (%)	4 (33.3)	6 (17.1)	0.237^b^
**Treatment**			
*n*	12	35	
Prednisone median in mg/day (IQR)	7.5 (39.4)	5.0 (10.0)	0.258^a^
Azathioprine, *n* (%)	3 (25.0)	15 (42.9)	0.272^b^
Chloroquine, *n* (%)	4 (33.3)	13 (37.1)	0.813^b^
Cyclophosphamide, *n* (%)	2 (16.7)	2 (5.7)	0.241^b^

*IQR, interquartile range; APS, seropositive antiphospholipid syndrome*.

*^a^Wilcoxon–Mann–Whitney test*.

*^b^Chi square test*. *Bold font indicates p < 0.05*.

### Handling of Serum Samples

Thirty nine serum samples from patients with SLE and 14 from NHD were used for phagocytosis assays in the presence of native serum. One aliquot of each serum was treated with heat (56°C) for 30 min for complement inactivation (28). Native and heat-inactivated samples were stored together at −20°C from the day of blood withdrawal and thawed once immediately before each phagocytosis assay. Additional 15 samples from patients with SLE and 4 from NHD were available for phagocytosis assays only in form of heat-inactivated serum.

### Measurement of Serum LPC

**Figure 1:**
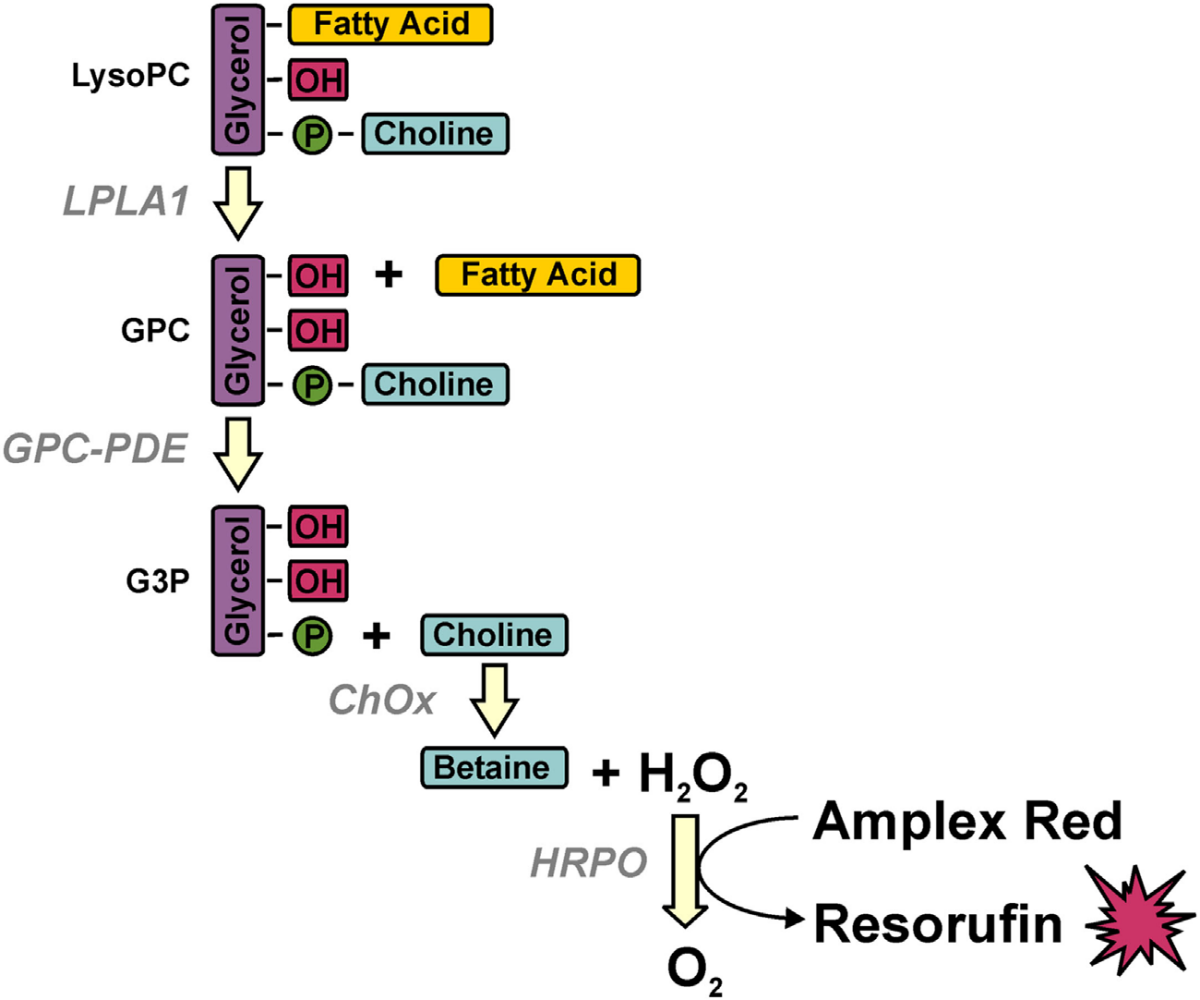
Multi-step coupled enzymatic test for the detection of lysophosphatidylcholine (LPC). LPC is stepwise converted to glycerophosphocholine, choline, and betaine, which eventually is detected in a peroxidase reaction with Amplex Red. Methodologically, baseline fluorescence is recorded by incubating the samples with all ingredients except for the first enzyme (lysophospholipase A1) whose addition defines the start of LPC conversion and the value of fluorescence, which is subtracted from the final value of fluorescence for calculating the amount of LPC on the basis of a standard curve of purified LPC.

Serum LPC concentrations were measured by a multi-step coupled enzymatic assay with slight modifications according to the protocol described (29). LPC was converted stepwise to glycerophosphocholine, choline, and betaine, which eventually was detected in a peroxidase reaction with Amplex Red (Figure 1). Briefly, 20 µl of diluted sera (1:100) were added to 96-well plates, 250 µl of prewarmed mix A consisting of 100 mM Tris–HCl pH 8.0, 0.01% Triton X-100, 1 mM calcium chloride, 20 µM Amplex Red, 1 U/ml horseradish peroxidase, 0.1 U/ml glycerophosphocholine phosphodiesterase, 1 U/ml choline oxidase (all from Sigma-Aldrich, Taufkirchen, Germany) were added, and baseline fluorescence was recorded for 15 min at 37°C in a Synergy Mx plate reader (BioTek Instruments, Bad Friedrichshall, Germany) at 563 nm excitation and 587 nm emission. Then, the reaction was started by adding 75 µl of mix B consisting of 100 mM Tris–HCl pH 8.0, 0.01% Triton X-100, and 20 µg/ml lysophospholipase A1 (cloned and purified from human THP-1 cells), and Amplex Red conversion was recorded over 40 min every minute. LPC concentrations were determined from the delta in fluorescence with a standard curve of purified LPC (Sigma-Aldrich).

### Necrotic Cells

Necrotic Raji cells served as target cells in our phagocytosis experiments. Viable Raji cells were labeled with carboxyfluorescein succinimidyl ester-diacetate (CFSE-DA, Molecular Probes, Eugene, OR, USA) 1 day prior to the phagocytosis experiments according to manufacturer instructions and kept at 37°C in 5.5% CO_2_ atmosphere in complete medium [RPMI 1640 medium containing 1% l-glutamine (Thermo Fisher Scientific, Munich, Germany)], 1% *N*-2-hydroxyethylpiperazine-*N*′-2-ethanesulfonic acid (HEPES; Merck, Darmstadt, Germany), and 1% penicillin–streptomycin (Thermo Fisher Scientific) supplemented with 10% fetal calf serum (FCS, Thermo Fisher Scientific). Necrosis was induced immediately before phagocytosis by incubation at 56°C for 30 min. Verification of necrosis was done by propidium iodide staining in phosphate-buffered saline (PBS) (Thermo Fisher Scientific). More than 99% of the cells were positive for propidium iodide.

### *In Vitro* Differentiation of Human Monocyte-Derived Macrophages (HMDM)

Peripheral blood mononuclear cells (PBMC) of three healthy volunteers (blood group 0) were isolated from venous heparinized blood by Ficoll density-gradient centrifugation (Lymphoflot; Biotest, Dreieich, Germany). Platelets were depleted by centrifugation through a cushion of FCS. Monocytes were separated from PBMC by magnetic cell sorting employing human CD14 MicroBeads following the manufacturer’s instructions (Miltenyi Biotec, Bergisch-Gladbach, Germany) and stained with the carbocyanine membrane dyes DiI or DiD (Thermo Fisher Scientific). Monocytes were differentiated to HMDM *in vitro* either at 50,000 cells/well (assays with patients’ sera) or 100,000 cells/well (LPC interference assay) for 7 days at 37°C and 5.5% CO_2_ humidified atmosphere in complete medium supplemented with 10% autologous serum and 10 ng/ml granulocyte-macrophage colony-stimulating factor (GM-CSF; Pharmaserv, Marburg, Germany). Fresh medium was added at days 3 and 5 after isolation.

### Phagocytosis of Necrotic Cells

Medium from wells containing stained HMDM was removed and replaced by 100,000 necrotic Raji cells suspended in 200 µl complete medium containing 10% FCS, 10% serum samples from SLE patients or healthy controls, 10% autologous serum, and varying concentrations of LPC (Sigma-Aldrich), respectively. Medium containing up to 0.7% ethanol as LPC-vehicle was used as control in phagocytosis assays with added LPC. Phagocytosis was allowed for 1 h at 37°C in 5.5% CO_2_ atmosphere. Adherent HMDM were detached by rinsing with cold PBS containing 2 mM ethylenediamine tetraacetic acid (EDTA; Merck).

### Flow Cytometry and Data Management

The uptake of necrotic cells by HMDM was monitored by two-color flow cytometry. HMDM were identified by size and by positive staining for DiI or DiD. HMDM being double-positive for CFSE and DiI or DiD, respectively, were considered to have ingested necrotic cells, which was further confirmed by confocal laser microscopy as reported previously (30). The phagocytosis index (PhIx) was calculated as the standardized product of the percentage of double-positive HMDM and the mean CFSE fluorescence intensity (relative amount of engulfed material) of double-positive HMDM. Experiments with patients’ sera were carried out in duplicates and assays with exogenously added LPC in triplicates. Standardization enabled fusion of results from different days and dyes and was achieved by normalizing the measured PhIx to phagocytosis in the absence of human serum of each experiment. In the LPC titration assays, normalization was done by defining the PhIx of the LPC-free samples as 100%.

### Statistical Analyses

Statistical analyses were performed using Prism 5 for Windows (GraphPad Software, Inc., La Jolla, CA, USA) or SPSS Statistics 21 (IBM Corp., Armonk, NY, USA), respectively. Correlations were calculated according to the non-parametric Spearman algorithm. For comparisons between groups, normal distribution was confirmed by Kolmogorov–Smirnov tests followed by two-sided Student’s *t*-tests. If normality was rejected, group comparisons were performed by two-sided Wilcoxon–Mann–Whitney tests. Independence of variables was examined by Chi square tests. Levels of *p* < 0.05 were considered as statistically significant.

## Results

In order to examine if SLE patients display alterations in LPC serum levels and if this might be of functional relevance for prey cell engulfment, we established a multi-step enzymatic test for the detection of LPC and applied the sera to *in vitro* phagocytosis assays with HMDM and dead Raji cells.

### SLE Patients Exhibit Increased Levels of Serum LPC

**Figure 2:**
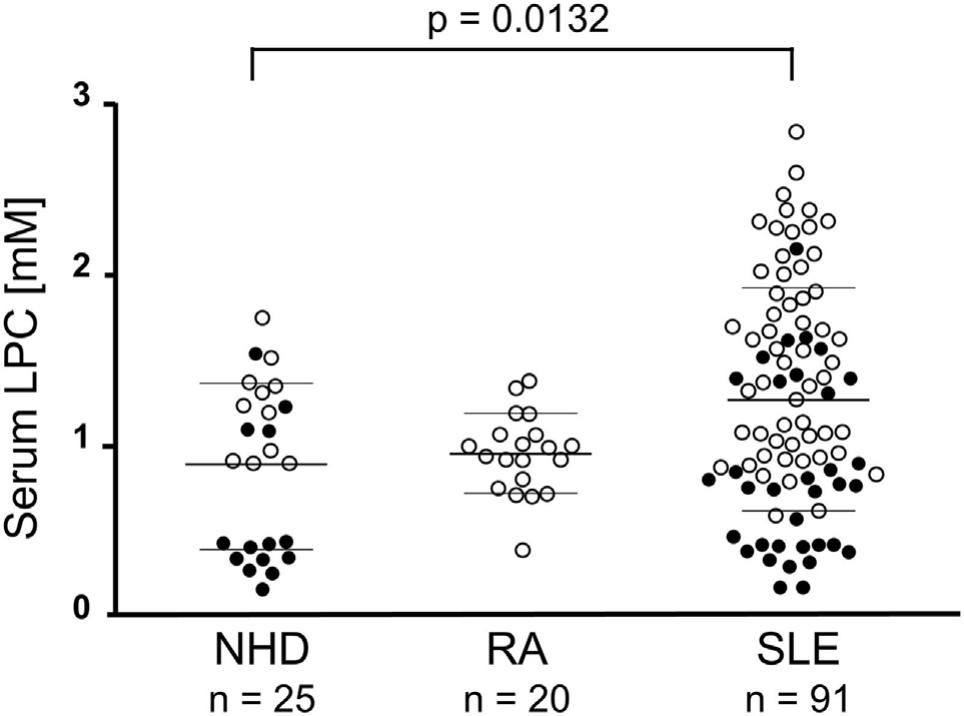
Lysophosphatidylcholine (LPC) serum levels of patients with systemic lupus erythematosus (SLE) are elevated. In serum samples of normal healthy donors (NHD), patients with rheumatoid arthritis (RA) and SLE LPC concentrations were analyzed by a multi-step coupled enzymatic test as depicted in Figure 1. LPC levels from individual serum samples, their means, and SDs are shown. Open symbols (only LPC values available), filled symbols (LPC values and phagocytosis index available), bold bars (cohort means), thin bars (cohort SDs).

The mean LPC serum concentration in the group of NHD as measured by the multi-step coupled enzymatic test was 886 ± 493.3 µM. In comparison, patients with SLE had significantly increased LPC serum levels (1,298 ± 687.9 µM; *p* = 0.0132; after Dunn’s correction *p* < 0.05). No significant differences were observed when comparing RA patients (953.1 ± 233.4 µM) with NHD or with SLE patients, respectively (Figure 2). To our knowledge, this is the first detailed report about concentrations of LPC in sera of patients with SLE.

### Demographical, Serological, and Clinical Characteristics of the SLE Cohort

**Figure 3:**
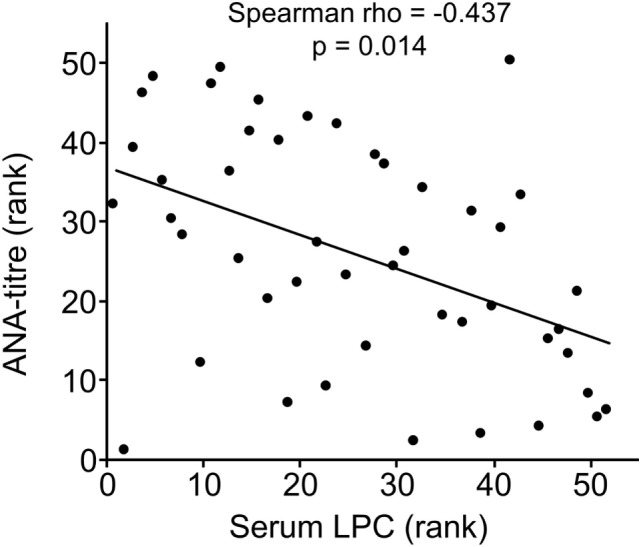
Lysophosphatidylcholine (LPC) serum levels of patients with systemic lupus erythematosus inversely correlated with antinuclear antibody (ANA) titers. Correlation analysis of serum LPC values and ANA titers. The values of ANA titers and serum LPC concentrations were transformed to rank values, and the Spearman rho correlation coefficient was calculated (*p* = 0.014 after Bonferroni correction).

Clinical and serological information about SLE patients are shown in Tables 1 and 2. Considering the actual clinical activity status of the patients, we observed that patients in flare (SLEDAI score ≥5 or ECLAM score ≥4, Table 2) showed significantly lower levels of serum complement C4 (in flare median: 10.7 mg/dl; no flare median 14.2 mg/dl; *p* = 0.038). The concentration of serum LPC was slightly elevated in patients with flares, but this difference did not reach statistical significance (not shown). We observed particularly high LPC serum levels in patients with vessel (*p* = 0.036) and kidney (*p* = 0.030) involvement (Table 3). Unfortunately, no complete data on renal function were available. Five out of 16 patients with renal involvement showed severely compromised renal function as manifested by high protein levels in urine and creatinine clearance lower than 50 ml/min (not shown), but no significant association with LPC serum levels was detected. Interestingly, serum LPC levels correlated significantly and inversely (*p* = 0.014) with antinuclear antibody (ANA) titers in patients with SLE (Figure 3). No other statistical association with laboratory parameters (Tables 1 and 2) or prednisone treatment was found (not shown).

**Table 3:** Serum concentrations of LPC and phagocytosis of necrotic cells in inactive serum stratified by organ involvement in patients with systemic lupus erythematosus.

Organ	Involvement	*n* (%)	LPC (µM) Mean ± SD	*p*	PhIx Mean ± SD	*p*
Vessels	Yes	6 (12.5)	**1,561.7 ± 519.0**	**0.036**	295.1 ± 104.4	0.051
	No	42 (87.5)	**952.2 ± 660.4**		427.8 ± 190.1	
Kidney	Yes	16 (33.3)	**1,322.4 ± 715.5**	**0.030**	371.3 ± 166.9	0.137
	No	32 (66.7)	**881.3 ± 607.0**		433.0 ± 194.8	
APS	Yes	10 (20.0)	1,091.1 ± 653.4	0.760	427.1 ± 119.8	0.386
	No	40 (80.0)	1,013.9 ± 672.8		407.6 ± 200.8	
Skin	Yes	25 (52.1)	1,107.9 ± 648.2	0.398	414.2 ± 178.2	0.459
	No	23 (47.9)	941.9 ± 699.3		408.6 ± 198.9	
Joints	Yes	18 (37.5)	998.1 ± 644.5	0.691	406.1 ± 192.1	0.351
	No	30 (62.5)	1,078.9 ± 729.7		414.5 ± 186.1	
Leukopenia	Yes	14 (29.2)	770.0 ± 490.4	0.088	477.2 ± 197.8	0.060
	No	34 (70.8)	1,134.6 ± 712.1		385.4 ± 177.5	
Central nervous system	Yes	6 (12.5)	945.7 ± 812.6	0.751	422.2 ± 130.2	0.448
	No	42 (87.5)	1,040.2 ± 659.4		410.4 ± 192.7	
Serosal membranes	Yes	11 (22.9)	926.2 ± 529.0	0.571	455.8 ± 247.3	0.189
	No	37 (77.1)	1,058.7 ± 711.5		398.8 ± 166.5	

*LPC, lysophosphatidylcholine; PhIx, phagocytic index; APS, seropositive anti-phospholipid syndrome*.

Bold font indicates p < 0.05

### Destruction of Heat-Labile Serum Components Reveals the Distinct Effect of Thermostable Factors on Phagocytosis of Necrotic Cells *In Vitro*

**Figure 4:**
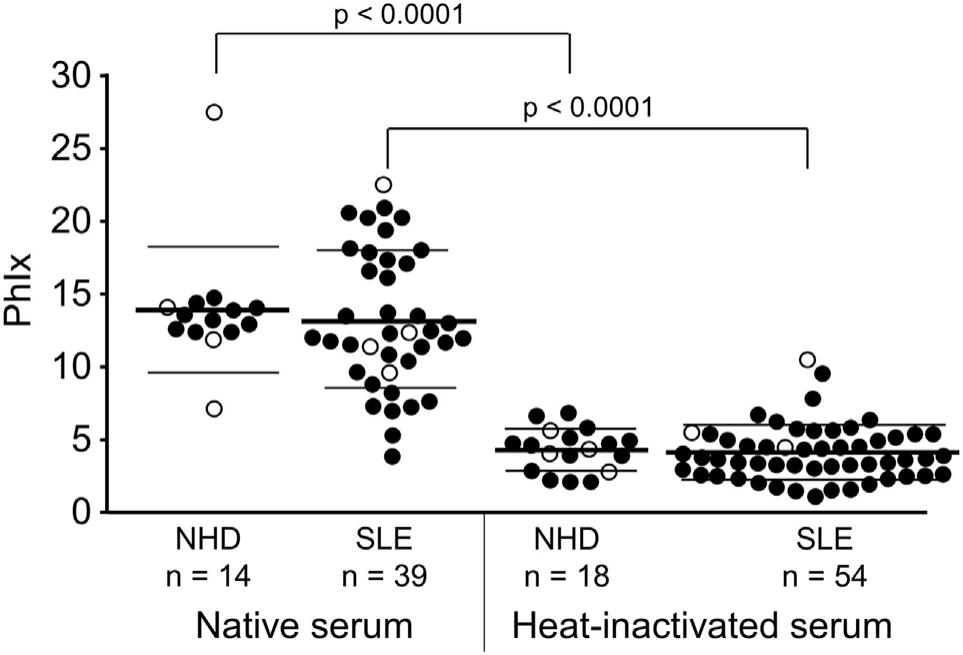
Influence of heat-labile serum components on the phagocytosis of necrotic cells by human monocyte-derived macrophages. Uptake of necrotic cells was analyzed by flow cytometry and quantified by the phagocytosis index (PhIx). Phagocytosis was allowed in the presence of native or heat-inactivated (56°C for 30 min) serum from normal healthy donors (NHD) or patients with systemic lupus erythematosus (SLE). Phagocytosis in heat-inactivated serum was lower in both cohorts than in native serum (NHD and SLE each *p* < 0.0001). There was no significant difference in phagocytosis between the NHD and SLE cohort neither in native serum nor in heat-inactivated serum. Open symbols (only PhIx available), filled symbols (PhIx and lysophosphatidylcholine concentration available), bold bars (cohort means), thin bars (cohort SDs).

In a first step to address the potential association of serum LPC levels with the process of dying cell clearance and SLE disease, we analyzed the effects of NHD and SLE sera in their native and heat-inactivated form on the phagocytosis of necrotic cells by HMDM (Figure 4). The uptake of necrotic cells was quantified by the PhIx. Phagocytosis in the presence of native sera from NHD (13.9 ± 4.3) was similar to that in the presence of native sera from patients with SLE (13.2 ± 4.7). In order to eliminate the influence of heat-labile serum factors on phagocytosis and to focus on the effects of thermally stable components (like LPC), sera were heat inactivated at 56°C for 30 min. This strongly reduced the uptake of necrotic cells in comparison to native serum in the NHD (*p* < 0.0001) as well as the SLE (*p* < 0.0001) cohort, respectively—most likely due to inactivation of complement and other heat-sensitive opsonins. Again, addition of inactivated sera from NHD (4.3 ± 1.5) and SLE patients (4.1 ± 1.9) resulted in similar levels of phagocytosis, and no significant difference in PhIx was observed.

### Serum LPC Levels of SLE Patients Negatively Correlate with Phagocytosis of Necrotic Cells *In Vitro*

**Figure 5:**
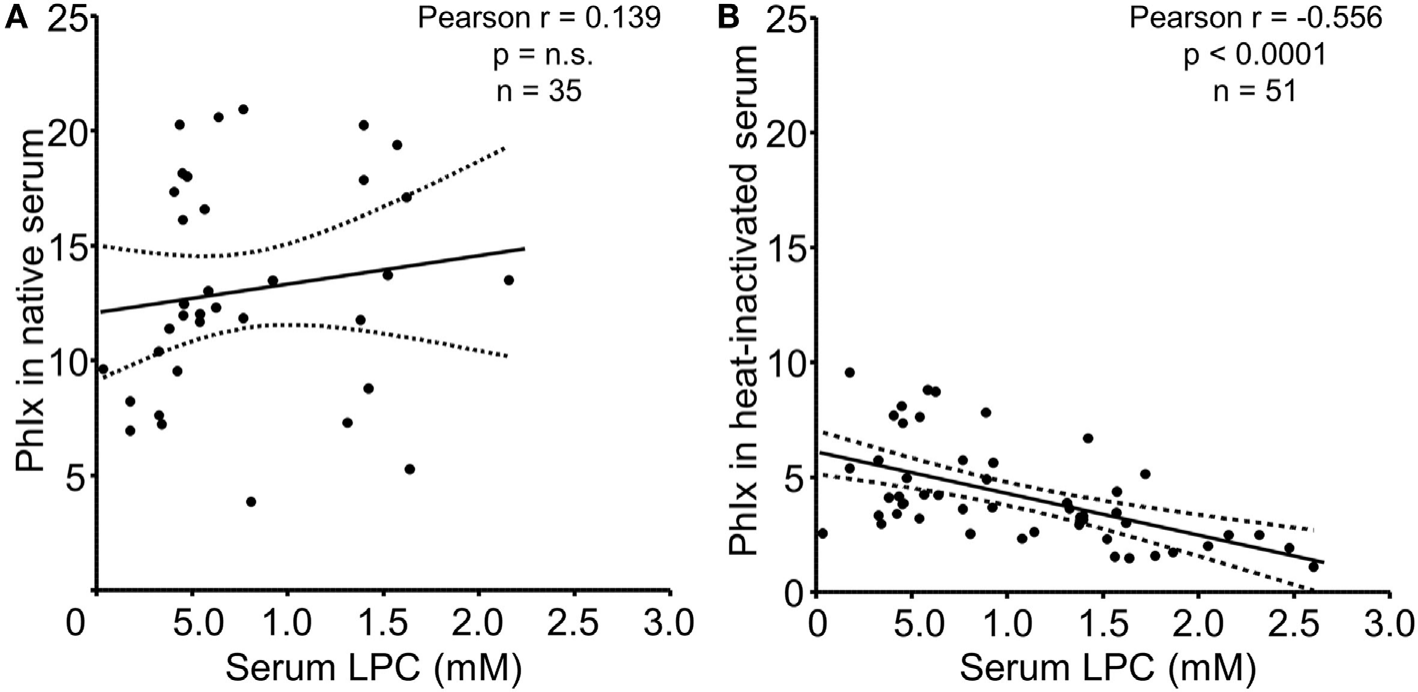
Serum lysophosphatidylcholine (LPC) levels of systemic lupus erythematosus (SLE) patients correlate negatively with phagocytosis of necrotic cells *in vitro*. Serum LPC levels of SLE patients were correlated to phagocytosis of necrotic cells in the presence of the respective serum as determined by the phagocytosis index (PhIx). Each data point represents an individual serum value. **(A)** Phagocytosis assays in the presence of native serum. n.s., not significant. **(B)** Phagocytosis assays in the presence of heat-inactivated serum. Dashed lines indicate the 95% confidence interval.

In the next step, we analyzed the relationship between serum LPC concentrations and phagocytosis as quantified by the PhIx. No significant correlation was observed in case of native sera, neither in the NHD (not shown) nor in the SLE group (Figure 5A). In line with our previous findings and reports by others, complement C4 and ANA titers revealed significant correlations with phagocytosis in native serum (not shown) (30). Upon heat inactivation, the impact of thermosensitive components, including complement, is eliminated, and the influence of thermostable serum factors on the engulfment process can be examined. LPC belongs to the group of thermostable serum components due to its lipid nature. Whereas phagocytosis in the presence of heat-inactivated serum from NHD showed no significant association with LPC concentrations (not shown), a highly significant negative correlation (*r* = –0.556, *p* < 0.0001) between LPC levels and phagocytosis in case of heat-inactivated serum from patients with SLE was observed (Figure 5B).

We also performed group comparisons for all organ involvement groups. Sera from patients with kidney or vessel involvement with particularly high LPC levels displayed a tendency toward reduced phagocytosis activity. However, this did not reach statistical significance. In turn, sera from patients with leukopenia showed particularly low LPC levels and a trend toward increased phagocytosis activity (Table 3). This underlines the negative relationship between LPC serum concentration and phagocytosis activity and raises the question of causality.

### Exogenously Added LPC Reduces Phagocytosis of Necrotic Cells *In Vitro*

**Figure 6:**
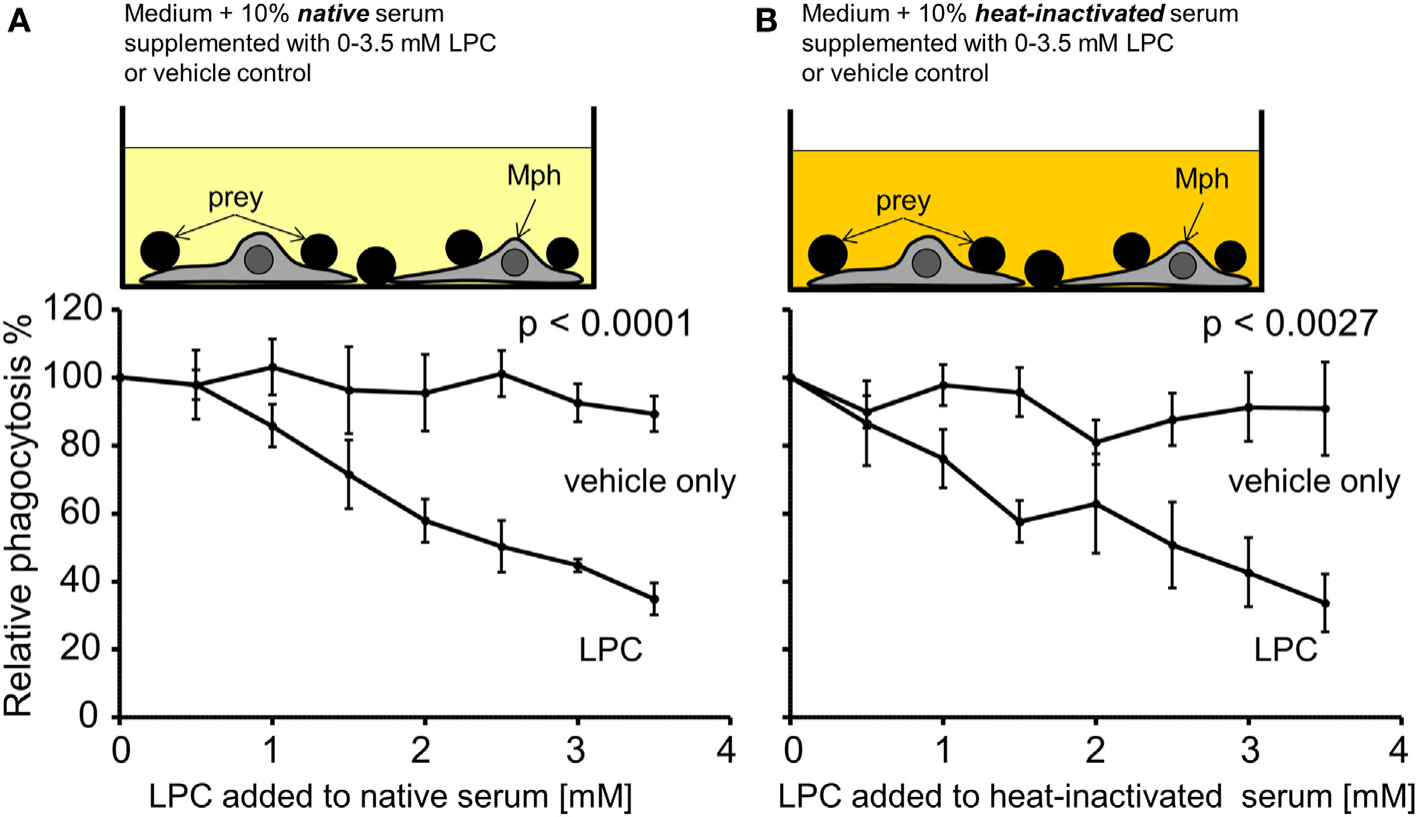
Exogenously added lysophosphatidylcholine (LPC) reduces phagocytosis of necrotic cells *in vitro*. Normal healthy donor serum was supplemented with the indicated concentrations of purified LPC, added to phagocytosis assays with necrotic cells in its native or heat-inactivated form, and phagocytosis was measured as % of unsupplemented serum (100% control). **(A)** Data for native serum. **(B)** Data for heat-inactivated serum. The addition of LPC resulted in a significant concentration-dependent decline in phagocytosis in comparison to the LPC-free control (two-way ANOVA test). Means ± SEs of triplicates are shown.

As a first approach to analyze the causality between high serum LPC levels and decreased uptake of necrotic cells, NHD serum was supplemented with increasing concentrations of purified LPC (in the range that was measured in the SLE sera). Figure 6 compares the phagocytosis results of LPC supplementation versus the corresponding vehicle control. All PhIxs were normalized to the PhIx value of the original serum (added LPC concentration = 0 mM). Addition of LPC resulted in a significant concentration-dependent decline in phagocytosis in native serum (*p* < 0.0001) as well as in heat-inactivated serum (*p* = 0.0027) in comparison to the LPC-free control (Figures 6A,B). The phagocytosis of necrotic cells was similarly inhibited by LPC to 35% in native and to 34% in heat-inactivated serum. The vehicle control showed no influence on phagocytosis, neither in native nor in heat-inactivated serum.

## Discussion

Billions of cells die in the context of normal tissue regeneration every day. If they are not cleared in time, the process of death enters the stage of secondary necrosis and SNEC accumulate in peripheral tissues (3). Pioneering experimental evidence for the existence of this kind of non-engulfed cellular debris in tissue samples from patients with SLE was already provided in the late 1950s (31). Several studies reported so-called hematoxylin bodies defined as exclusively extracellular, single, clustered, or coalescent corpses found specifically in tissues of 70–90% of virtually untreated patients with SLE (32–35). Regrettably, at that time, these alterations could not be associated with cell death phenomena, because apoptosis was firstly described much later (36). More recently, increased amounts of apoptotic PBMC and neutrophils have been reported in patients with SLE (37, 38). The process of apoptosis itself turned out not to be affected (39). Instead, an engulfment dysfunction was identified as the underlying reason for the accumulation of uncleared, dead cell material in patients with SLE (40).

In the course of apoptosis, the plasma membrane of cells undergoes characteristic changes. Whereas viable cells exhibit a clear membrane phospholipid asymmetry, during apoptosis, redistribution of phospholipids between the membrane leaflets occurs (41, 42). Moreover, phospholipases (PLA_2_) generate LPC by hydrolyzing the fatty acid at the sn-2 position of glycerophospholipids (43–45). Calcium-independent phospholipase A2 (iPLA_2_) is processed during apoptosis by caspase-3-mediated cleavage leading to a truncated form with increased activity (46), and LPC appears to be the major product of lipid catabolism in apoptotic cells (45, 47). Its release under participation of the membrane transporter ABCA1 and its role as chemoattractant in the clearance of dead cells has been confirmed by us and others (18, 21, 24).

In the present study, we observed increased LPC levels in sera of patients with SLE, and the highest levels were detected in patients with vessel or kidney involvement (Figure 2). In principle, elevated serum LPC levels could be a cause as well as a consequence of SLE disease. It is feasible to assume that accumulating dying cell material due to known clearance deficiencies in SLE could give rise to accumulation of LPC in organs and tissues, which might finally “spill over” into the serum. This might be particularly strong in case of vasculitis since, here, cell death occurs in very close proximity to the blood. In case of kidney involvement, renal dysfunction and impaired lipid clearance might additionally contribute to the increase in LPC serum levels as has been reported in the context of type II diabetes and renal transplantation (48, 49). Vice versa, elevated LPC serum levels could affect dying cell clearance by interfering with proper phagocyte recruitment leading to delays in dying cell clearance and establishment as well as maintenance of SLE disease.

Lysophosphatidylcholine is a potent find-me signal of dying cells (50–53), provided that a concentration gradient between the dying/dead cell and the phagocyte can be established. Experimental neutralization of this gradient by adding exogenous LPC can efficiently interfere with chemoattraction of THP1-monocytes by supernatants of apoptotic cells (21), and sera from patients with SLE reportedly reduce chemoattraction of macrophages by supernatants of apoptotic cells (54). In contrast, NHD sera or sera from RA patients show no inhibition of phagocyte migration (54). Hence, the elevated LPC levels in SLE sera reported here might be the reason for the inhibition of chemoattraction in our previous study (54). Along the same lines, we now observed a significant correlation between high LPC levels in SLE sera and interference with phagocytosis of dead cells, but only in case of heat-inactivated sera, where the influence of ANAs, antiphospholipid antibodies, complement factors, and other heat-labile opsonins has been minimized (Figure 5B). ANAs, antiphospholipid antibodies, and complement factors are well-known to affect the internalization of dying cells, and their titers can vary strongly in sera of SLE patients (55–58). In turn, heat-inactivation of serum allows studying the phagocytic impact of heat-stable compounds, including LPC. This might be specifically relevant in tissues where the concentration of serum proteins is low. It should be noted that we observed this correlation between high LPC serum levels and interference with phagocytosis of dead cells only in SLE but not in NHD sera. Further subgroup analyses revealed that the correlation was particularly strong for LPC serum levels >1 mM (not shown). This might explain why no significant correlation for NHD sera (mean LPC concentration 886 ± 493.3 µM) was observed and suggests that a certain type of threshold does exist in this regard. Detailed analyses of the groups with different organ involvements revealed trends of high serum LPC levels and low phagocytosis activity in case of kidney and vessel involvement as well as low serum LPC level and high phagocytosis activity in case of leukopenia. Although these trends did not reach statistical significance, they argue in favor of a negative relationship between increased serum LPC concentrations and phagocytic activity.

Evidence for the causal involvement of LPC in phagocytosis inhibition derives from our add-in experiments: NHD serum supplemented with increasing concentrations of purified LPC hampered dead cell phagocytosis in an LPC concentration-dependent manner irrespective if it was heat-inactivated or not (Figure 6). Of note, the add-in phagocytosis experiments were performed with NHD serum which—in contrast to SLE sera—is of rather constant composition and lacks relevant titers of autoantibodies. This represents a plausible explanation why addition of LPC to native and inactivated NHD serum had similar effects on phagocytosis, while the correlation between LPC levels in SLE sera and phagocytic activity was only measurable upon heat inactivation.

Interestingly, we observed higher LPC serum levels in patients with flare than patients with non-flare. Although this trend was statistically not significant, it might be speculated that elevated levels of LPC function as a clearance inhibitor at a cellular level and lead to SLE disease exacerbation ensuing from reduced efferocytosis, augmented release of autoantigens, and immune complex formation, and resulting in enforced inflammatory conditions and clinical symptoms.

**Figure 7:**
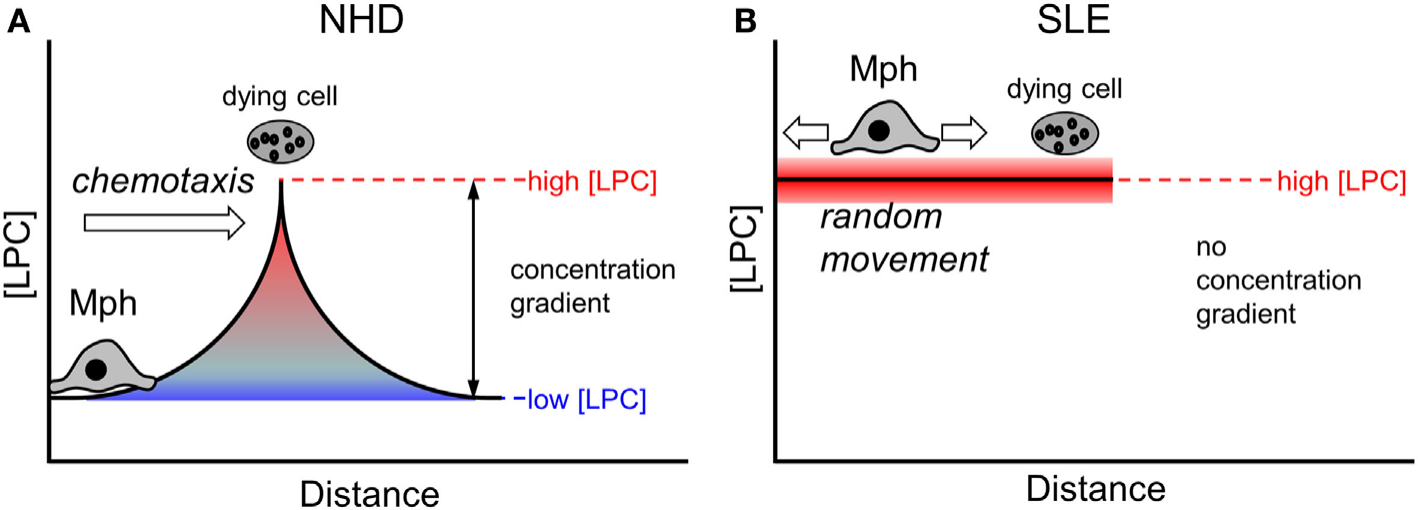
Effect of abnormal high lysophosphatidylcholine (LPC) levels on chemotaxis of macrophages. A local chemotactic concentration gradient is required for the attraction and migration of phagocytes toward dying and dead cells. Under non-pathological conditions, an LPC concentration gradient is formed around a dying cell **(A)**. Macrophages follow the gradient until they encounter the dying cell and then start the phagocytic process. In conditions with increased extracellular LPC concentrations, like we observed in systemic lupus erythematosus (SLE) sera, the amount of LPC released by the dying cell might not be high enough to establish a functional chemotactic gradient **(B)**. Macrophages cannot be attracted, they move randomly. The likelihood of contacts between macrophages and dying cells is reduced resulting in decreased phagocytosis.

Eat-me signals presented on the membrane surface require a direct cell–cell contact in order to be functional. In contrast, find-me signals are released from dying and dead cells and operate over a certain distance [reviewed in Ref. (59)]. The existence of a chemotactic concentration gradient is a prerequisite for the attraction and migration of phagocytes. Under non-pathological conditions (as is the case in the presence of NHD sera), local gradients of find-me signals can be successfully built up around a dying cell (Figure 7A). Macrophages follow the gradient until they encounter dying cells and then can recognize and engulf them *via* exposed eat-me signals. In conditions of abnormally high LPC concentrations in the extracellular milieu (as we observed in SLE sera and upon supplementation of NHD sera in our phagocytosis assays), the LPC amount that is locally released by dying and dead cells appears not to be sufficient to establish an effective chemotactic gradient (Figure 7B). Consequently, macrophages cannot follow the gradient and do not find their way to the dying prey. Our data suggest that impaired chemoattraction decreases the likelihood of macrophages meeting dead cells, thus resulting in the observed decrease in phagocytosis.

Overall, our study is the first to provide experimental evidence for increased serum LPC levels in SLE patients. *In vitro*, this correlated with impaired phagocytosis of dead cells when the respective SLE sera were added to phagocytosis assays and when NHD sera were supplemented with LPC concentrations in the range as measured in SLE sera. Accordingly, we suggest that high extracellular LPC levels can neutralize local LPC gradients, which are required for phagocyte attraction in the context of dying cell clearance, thus contributing to the establishment and/or perpetuation of SLE disease and rendering serum LPC a potential, mechanisms-based marker of SLE disease, which deserves further validation.

## Ethics Statement

This study was carried out in accordance with the recommendations of the ethics committee of the Friedrich-Alexander University Erlangen with written informed consent from all subjects. All subjects gave written informed consent in accordance with the Declaration of Helsinki. The protocol was approved by the ethics committee of the Friedrich-Alexander University Erlangen (Permit number 52_14 B).

## Author Contributions

GG, LM, KL, and MH conceived and designed the study. GG, HK, CJ, LM, and KL performed experiments and analyzed the data. JR collected patients samples and clinical data. SB re-evaluated cases and analyzed the data LM, GG, and KL wrote the manuscript. All authors reviewed the manuscript.

## Conflict of Interest Statement

The authors declare that the research was conducted in the absence of any commercial or financial relationships that could be construed as a potential conflict of interest.

## References

[1] Elliott MR Ravichandran KS. The dynamics of apoptotic cell clearance. Dev Cell (2016) 38(2):147–60. 10.1016/j.devcel.2016.06.029 27459067 PMC4966906

[2] Rekvig OP Van der Vlag J The pathogenesis and diagnosis of systemic lupus erythematosus: still not resolved. Semin Immunopathol (2014) 36(3):301–11. 10.1007/s00281-014-0428-6 24763531

[3] Munoz LE Lauber K Schiller M Manfredi AA Herrmann M. The role of defective clearance of apoptotic cells in systemic autoimmunity. Nat Rev Rheumatol (2010) 6(5):280–9. 10.1038/nrrheum.2010.46 20431553

[4] Munoz LE Berens C Lauber K Gaipl US Herrmann M Apoptotic cell clearance and its role in the origin and resolution of chronic inflammation. Front Immunol (2015) 6:139 10.3389/fimmu.2015.00139 25859248 PMC4373391

[5] Mahajan A Herrmann M Munoz LE. Clearance deficiency and cell death pathways: a model for the pathogenesis of SLE. Front Immunol (2016) 7:35. 10.3389/fimmu.2016.00035 26904025 PMC4745266

[6] Cervera R Khamashta MA Font J Sebastiani GD Gil A Lavilla P Morbidity and mortality in systemic lupus erythematosus during a 10-year period: a comparison of early and late manifestations in a cohort of 1,000 patients. Medicine (2003) 82(5):299–308. 10.1097/01.md.0000091181.93122.55 14530779

[7] Baumann I Kolowos W Voll RE Manger B Gaipl U Neuhuber WL Impaired uptake of apoptotic cells into tingible body macrophages in germinal centers of patients with systemic lupus erythematosus. Arthritis Rheum (2002) 46(1):191–201. 10.1002/1529-0131(200201)46:1<191::AID-ART10027>3.0.CO;2-K 11817590

[8] Sherer Y Gorstein A Fritzler MJ Shoenfeld Y. Autoantibody explosion in systemic lupus erythematosus: more than 100 different antibodies found in SLE patients. Semin Arthritis Rheum (2004) 34(2):501–37. 10.1016/j.semarthrit.2004.07.002 15505768

[9] Casciola-Rosen LA Anhalt G Rosen A. Autoantigens targeted in systemic lupus erythematosus are clustered in two populations of surface structures on apoptotic keratinocytes. J Exp Med (1994) 179(4):1317–30. 10.1084/jem.179.4.1317 7511686 PMC2191465

[10] Munoz LE Janko C Grossmayer GE Frey B Voll RE Kern P Remnants of secondarily necrotic cells fuel inflammation in systemic lupus erythematosus. Arthritis Rheum (2009) 60(6):1733–42. 10.1002/art.24535 19479824

[11] Biermann MH Veissi S Maueroder C Chaurio R Berens C Herrmann M The role of dead cell clearance in the etiology and pathogenesis of systemic lupus erythematosus: dendritic cells as potential targets. Expert Rev Clin Immunol (2014) 10(9):1151–64. 10.1586/1744666x.2014.944162 25081199

[12] Pons-Estel GJ Alarcon GS Scofield L Reinlib L Cooper GS. Understanding the epidemiology and progression of systemic lupus erythematosus. Semin Arthritis Rheum (2010) 39(4):257–68. 10.1016/j.semarthrit.2008.10.007 19136143 PMC2813992

[13] Chakravarty EF Bush TM Manzi S Clarke AE Ward MM Prevalence of adult systemic lupus erythematosus in California and Pennsylvania in 2000: estimates obtained using hospitalization data. Arthritis Rheum (2007) 56(6):2092–4. 10.1002/art.22641 17530651 PMC2530907

[14] Lawrence RC Helmick CG Arnett FC Deyo RA Felson DT Giannini EH Estimates of the prevalence of arthritis and selected musculoskeletal disorders in the United States. Arthritis Rheum (1998) 41(5):778–99. 10.1002/1529-0131(199805)41:5<778::AID-ART4>3.0.CO;2-V 9588729

[15] Lahita RG Emerging concepts for sexual predilection in the disease systemic lupus erythematosus. Ann N Y Acad Sci (1999) 876:64–9; discussion 9–70. 10.1111/j.1749-6632.1999.tb07623.x 10415594

[16] Bengtsson AA Ronnblom L. Systemic lupus erythematosus: still a challenge for physicians. J Intern Med (2017) 281(1):52–64. 10.1111/joim.12529 27307107

[17] Hochberg MC Updating the American College of Rheumatology revised criteria for the classification of systemic lupus erythematosus. Arthritis Rheum (1997) 40(9):1725 10.1002/art.1780400928 9324032

[18] Lauber K Bohn E Krober SM Xiao YJ Blumenthal SG Lindemann RK Apoptotic cells induce migration of phagocytes via caspase-3-mediated release of a lipid attraction signal. Cell (2003) 113(6):717–30. 10.1016/S0092-8674(03)00422-7 12809603

[19] Mueller RB Sheriff A Gaipl US Wesselborg S Lauber K. Attraction of phagocytes by apoptotic cells is mediated by lysophosphatidylcholine. Autoimmunity (2007) 40(4):342–4. 10.1080/08916930701356911 17516225

[20] Peter C Waibel M Keppeler H Lehmann R Xu G Halama A Release of lysophospholipid ‘find-me’ signals during apoptosis requires the ATP-binding cassette transporter A1. Autoimmunity (2012) 45(8):568–73. 10.3109/08916934.2012.719947 22913458

[21] Peter C Waibel M Radu CG Yang LV Witte ON Schulze-Osthoff K Migration to apoptotic “find-me” signals is mediated via the phagocyte receptor G2A. J Biol Chem (2008) 283(9):5296–305. 10.1074/jbc.M706586200 18089568

[22] Radu CG Yang LV Riedinger M Au M Witte ON. T cell chemotaxis to lysophosphatidylcholine through the G2A receptor. Proc Natl Acad Sci U S A (2004) 101(1):245–50. 10.1073/pnas.2536801100 14681556 PMC314170

[23] Wang L Radu CG Yang LV Bentolila LA Riedinger M Witte ON. Lysophosphatidylcholine-induced surface redistribution regulates signaling of the murine G protein-coupled receptor G2A. Mol Biol Cell (2005) 16(5):2234–47. 10.1091/mbc.E04-12-1044 15728718 PMC1087231

[24] Yang LV Radu CG Wang L Riedinger M Witte ON. Gi-independent macrophage chemotaxis to lysophosphatidylcholine via the immunoregulatory GPCR G2A. Blood (2005) 105(3):1127–34. 10.1182/blood-2004-05-1916 15383458

[25] Le LQ Kabarowski JH Weng Z Satterthwaite AB Harvill ET Jensen ER Mice lacking the orphan G protein-coupled receptor G2A develop a late-onset autoimmune syndrome. Immunity (2001) 14(5):561–71. 10.1016/S1074-7613(01)00145-5 11371358

[26] Lauber K Keppeler H Munoz LE Koppe U Schroder K Yamaguchi H Milk fat globule-EGF factor 8 mediates the enhancement of apoptotic cell clearance by glucocorticoids. Cell Death Differ (2013) 20(9):1230–40. 10.1038/cdd.2013.82 23832117 PMC3741508

[27] Aletaha D Neogi T Silman AJ Funovits J Felson DT Bingham CO III 2010 rheumatoid arthritis classification criteria: an American College of Rheumatology/European League Against Rheumatism collaborative initiative. Arthritis Rheum (2010) 62(9):2569–81. 10.1002/art.27584 20872595

[28] Linscott WD Triglia RP. The bovine complement system. Adv Exp Med Biol (1981) 137:413–30. 7331946

[29] Mancini A Del Rosso F Roberti R Caligiana P Vecchini A Binaglia L. Quantitation of glycerophosphorylcholine by flow injection analysis using immobilized enzymes. Mol Cell Biochem (1996) 162(2):83–7. 10.1007/BF00227533 8905629

[30] Grossmayer GE Munoz LE Weber CK Franz S Voll RE Kern PM IgG autoantibodies bound to surfaces of necrotic cells and complement C4 comprise the phagocytosis promoting activity for necrotic cells of systemic lupus erythaematosus sera. Ann Rheum Dis (2008) 67(11):1626–32. 10.1136/ard.2007.081828 18165321

[31] Munoz LE Leppkes M Fuchs TA Hoffmann M Herrmann M Missing in action – the meaning of cell death in tissue damage and inflammation. Immunol Rev (2017) 280(1):26–40. 10.1111/imr.12569 29027227

[32] Gross L The cardiac lesions in Libman-Sacks disease: with a consideration of its relationship to acute diffuse lupus erythematosus. Am J Pathol (1940) 16(4):375–408.11. 19970511 PMC1965099

[33] Gueft B Laufer A Further cytochemical studies in systemic lupus erythematosus. AMA Arch Pathol (1954) 57(3):201–26. 13137680

[34] Klemperer P Gueft B Lee S Leuchtenberger C Pollister A Cytochemical changes of acute lupus erythematosus. Arch Pathol (1950) 49(5):503–16.

[35] Worthington JW Jr Baggenstoss AH Hargraves MM Significance of hematoxylin bodies in the necropsy diagnosis of systemic lupus erythematosus. Am J Pathol (1959) 35:955–69. 13846002 PMC1934840

[36] Kerr JF Wyllie AH Currie AR. Apoptosis: a basic biological phenomenon with wide-ranging implications in tissue kinetics. Br J Cancer (1972) 26(4):239–57. 10.1038/bjc.1972.33 4561027 PMC2008650

[37] Courtney PA Crockard AD Williamson K Irvine AE Kennedy RJ Bell AL. Increased apoptotic peripheral blood neutrophils in systemic lupus erythematosus: relations with disease activity, antibodies to double stranded DNA, and neutropenia. Ann Rheum Dis (1999) 58(5):309–14. 10.1136/ard.58.5.309 10225817 PMC1752888

[38] Perniok A Wedekind F Herrmann M Specker C Schneider M. High levels of circulating early apoptic peripheral blood mononuclear cells in systemic lupus erythematosus. Lupus (1998) 7(2):113–8. 10.1191/096120398678919804 9541096

[39] Muñoz LE Frey B Appelt U Janko C Sarter K Voll RE Peripheral blood stem cells of patients with systemic lupus erythematosus show altered differentiation into macrophages. Open Autoimmun J (2010) 2:11–6. 10.2174/1876894601002010011

[40] Herrmann M Voll RE Zoller OM Hagenhofer M Ponner BB Kalden JR. Impaired phagocytosis of apoptotic cell material by monocyte-derived macrophages from patients with systemic lupus erythematosus. Arthritis Rheum (1998) 41(7):1241–50. 10.1002/1529-0131(199807)41:7<1241:aid-art15>3.0.co;2-h 9663482

[41] Martin SJ Reutelingsperger CP McGahon AJ Rader JA van Schie RC LaFace DM Early redistribution of plasma membrane phosphatidylserine is a general feature of apoptosis regardless of the initiating stimulus: inhibition by overexpression of Bcl-2 and Abl. J Exp Med (1995) 182(5):1545–56. 10.1084/jem.182.5.1545 7595224 PMC2192182

[42] Verhoven B Schlegel RA Williamson P. Mechanisms of phosphatidylserine exposure, a phagocyte recognition signal, on apoptotic T lymphocytes. J Exp Med (1995) 182(5):1597–601. 10.1084/jem.182.5.1597 7595231 PMC2192221

[43] Atsumi G Murakami M Tajima M Shimbara S Hara N Kudo I. The perturbed membrane of cells undergoing apoptosis is susceptible to type II secretory phospholipase A2 to liberate arachidonic acid. Biochim Biophys Acta (1997) 1349(1):43–54. 10.1016/S0005-2760(97)00082-9 9421195

[44] Hack CE Wolbink GJ Schalkwijk C Speijer H Hermens WT van den Bosch H A role for secretory phospholipase A2 and C-reactive protein in the removal of injured cells. Immunol Today (1997) 18(3):111–5. 10.1016/S0167-5699(97)01002-5 9078682

[45] Balsinde J Perez R Balboa MA. Calcium-independent phospholipase A2 and apoptosis. Biochim Biophys Acta (2006) 1761(11):1344–50. 10.1016/j.bbalip.2006.07.013 16962822

[46] Atsumi G Murakami M Kojima K Hadano A Tajima M Kudo I. Distinct roles of two intracellular phospholipase A2s in fatty acid release in the cell death pathway. Proteolytic fragment of type IVA cytosolic phospholipase A2alpha inhibits stimulus-induced arachidonate release, whereas that of type VI Ca2+-independent phospholipase A2 augments spontaneous fatty acid release. J Biol Chem (2000) 275(24):18248–58. 10.1074/jbc.M000271200 10747887

[47] Perez R Melero R Balboa MA Balsinde J. Role of group VIA calcium-independent phospholipase A2 in arachidonic acid release, phospholipid fatty acid incorporation, and apoptosis in U937 cells responding to hydrogen peroxide. J Biol Chem (2004) 279(39):40385–91. 10.1074/jbc.M402562200 15252038

[48] Lin J Hu FB Rimm EB Rifai N Curhan GC. The association of serum lipids and inflammatory biomarkers with renal function in men with type II diabetes mellitus. Kidney Int (2006) 69(2):336–42. 10.1038/sj.ki.5000021 16408124 PMC1630638

[49] Stojanovic D Cvetkovic T Stojanovic M Bojanic V Stefanovic N Radenkovic S Crosstalk of inflammatory mediators and lipid parameters as early markers of renal dysfunction in stable renal transplant recipients with regard to immunosuppression. Ann Transplant (2013) 18:414–23. 10.12659/AOT.889239 23946969

[50] Quinn MT Parthasarathy S Steinberg D. Lysophosphatidylcholine: a chemotactic factor for human monocytes and its potential role in atherogenesis. Proc Natl Acad Sci U S A (1988) 85(8):2805–9. 10.1073/pnas.85.8.2805 3357891 PMC280088

[51] Quinn MT Kondratenko N Parthasarathy S. Analysis of the monocyte chemotactic response to lysophosphatidylcholine: role of lysophospholipase C. Biochim Biophys Acta (1991) 1082(3):293–302. 10.1016/0005-2760(91)90205-V 2029549

[52] Ryborg AK Deleuran B Thestrup-Pedersen K Kragballe K. Lysophosphatidylcholine: a chemoattractant to human T lymphocytes. Arch Dermatol Res (1994) 286(8):462–5. 10.1007/BF00371572 7864659

[53] Hoffman RD Kligerman M Sundt TM Anderson ND Shin HS. Stereospecific chemoattraction of lymphoblastic cells by gradients of lysophosphatidylcholine. Proc Natl Acad Sci U S A (1982) 79(10):3285–9. 10.1073/pnas.79.10.3285 6954479 PMC346400

[54] Gaipl US Munoz LE Grossmayer G Lauber K Franz S Sarter K Clearance deficiency and systemic lupus erythematosus (SLE). J Autoimmun (2007) 28(2–3):114–21. 10.1016/j.jaut.2007.02.005 17368845

[55] Andreoli L Fredi M Nalli C Franceschini F Meroni PL Tincani A. Antiphospholipid antibodies mediate autoimmunity against dying cells. Autoimmunity (2013) 46(5):302–6. 10.3109/08916934.2013.783025 23713583

[56] Rovere P Manfredi AA Vallinoto C Zimmermann VS Fascio U Balestrieri G Dendritic cells preferentially internalize apoptotic cells opsonized by anti-beta2-glycoprotein I antibodies. J Autoimmun (1998) 11(5):403–11. 10.1006/jaut.1998.0224 9802923

[57] Kruse K Janko C Urbonaviciute V Mierke CT Winkler TH Voll RE Inefficient clearance of dying cells in patients with SLE: anti-dsDNA autoantibodies, MFG-E8, HMGB-1 and other players. Apoptosis (2010) 15(9):1098–113. 10.1007/s10495-010-0478-8 20198437

[58] Verbovetski I Bychkov H Trahtemberg U Shapira I Hareuveni M Ben-Tal O Opsonization of apoptotic cells by autologous iC3b facilitates clearance by immature dendritic cells, down-regulates DR and CD86, and up-regulates CC chemokine receptor 7. J Exp Med (2002) 196(12):1553–61. 10.1084/jem.20020263 12486098 PMC2196062

[59] Ravichandran KS. Find-me and eat-me signals in apoptotic cell clearance: progress and conundrums. J Exp Med (2010) 207(9):1807–17. 10.1084/jem.20101157 20805564 PMC2931173

